# Low‐Molecular‐Weight Heparin for Adult ICU Patients Who Require Thromboprophylaxis: Protocol for the INCEPT‐Thromboprophylaxis Platform Trial Domain

**DOI:** 10.1111/aas.70199

**Published:** 2026-01-30

**Authors:** Ruben Eck, Anders Granholm, Aske Tøgern, Aksel Karl Georg Jensen, Sandra Jonmarker, Fredrik Sjövall, Karina Meijer, Maria Cronhjort, Morten Hylander Møller, Anders Perner, Frederik Keus

**Affiliations:** ^1^ Department of Internal Medicine University Medical Center Groningen, University of Groningen Groningen the Netherlands; ^2^ Collaboration for Research in Intensive Care (CRIC) Copenhagen Denmark; ^3^ Department of Clinical Medicine, Faculty of Health Sciences University of Copenhagen Copenhagen Denmark; ^4^ Department of Intensive Care Copenhagen University Hospital—Rigshospitalet Copenhagen Denmark; ^5^ Section of Biostatistics, Department of Public Health University of Copenhagen Copenhagen Denmark; ^6^ Department of Clinical Sciences and Education Karolinska Institutet, Södersjukhuset Stockholm Sweden; ^7^ Department of Intensive and Perioperative Care Skane University Hospital Malmö Sweden; ^8^ Department of Clinical Sciences Malmö Lund University Lund Sweden; ^9^ Department of Hematology University Medical Center Groningen, University of Groningen Groningen the Netherlands; ^10^ Department of Clinical Sciences, Danderyd Hospital Karolinska Institutet Stockholm Sweden; ^11^ Department of Critical Care University Medical Center Groningen, University of Groningen Groningen the Netherlands

**Keywords:** adaptive platform trial, intensive care, LMWH, randomised clinical trial, thromboprophylaxis, trial protocol

## Abstract

**Background:**

Low‐molecular‐weight heparin (LMWH) is recommended for thromboprophylaxis in adult intensive care unit (ICU) patients. Despite its widespread use, there is insufficient evidence on the optimal dose, and there appears to be practice variation. Determining the LMWH dosing strategy that most effectively balances the risks of venous thromboembolism (VTE) and bleeding may improve outcomes in ICU patients.

**Methods:**

The *INCEPT‐thromboprophylaxis* domain is an investigator‐initiated, open‐label domain with an integrated feasibility phase on the international, pragmatic, parallel‐group, randomised, embedded, multifactorial, adaptive *Intensive Care Platform Trial (INCEPT)*. Adult acutely admitted ICU patients with an indication for thromboprophylaxis will be randomised to a fixed low dose, a fixed intermediate dose or a weight‐adjusted dose of LMWH while in the ICU for a maximum of 90 days. The primary outcome is days alive out of hospital at 30 days. Secondary outcomes include 30‐, 90‐ and 180‐day all‐cause mortality; days alive without life support at 30 and 90 days; days alive out of hospital at 90 days; days free of delirium at 30 days; health‐related quality of life and cognitive function at 180 days; VTE and major bleeding during hospital stay at 30 and 90 days; and serious adverse reactions at 30 and 90 days. Analyses will primarily be conducted in the intention‐to‐treat population using Bayesian statistical models with neutral, weakly informative priors. We will assess feasibility after 300 participants and conduct adaptive analyses after follow‐up for the primary outcome of 1500 participants and every additional 500 participants to a maximum of 10,000, with adaptive stopping for superiority/inferiority and practical equivalence (mean difference in the primary outcome < 1 day), and adaptive arm‐dropping for superiority/inferiority. Allocation will initially be equal, followed by response‐adaptive randomisation with a minimum 25% allocation to each arm. Expected sample sizes across different scenarios range from 2265 to 4892 participants, with approximately 100% probabilities of conclusiveness across scenarios.

**Conclusions:**

*INCEPT‐thromboprophylaxis* will with high probability provide conclusive results and inform clinical practice regarding the dosing of LMWH for thromboprophylaxis in adult acutely admitted ICU patients.

## 1 Introduction

Venous thromboembolism (VTE) can be a severe complication of critical illness. VTE is associated with acute adverse outcomes such as sudden clinical deterioration, and longer‐term sequelae including post‐thrombotic syndrome and chronic thromboembolic pulmonary hypertension [1, 2, 3]. VTE has a multifactorial origin and typically results from a combination of factors that predispose to endothelial damage, stasis of blood and a procoagulant state, including sepsis, trauma and surgery [4]. Intensive care unit (ICU) patients are commonly exposed to multiple risk factors, and despite routine thromboprophylaxis with low‐molecular‐weight heparin (LMWH) or, less commonly, unfractionated heparin, they face a residual VTE risk estimated between 3% and 26% in most cohort studies [4]. This suggests that higher LMWH doses might be beneficial. Conversely, ICU patients also face a bleeding risk estimated between 3% and 10% [5, 6, 7]. Thromboprophylaxis therefore requires striking the right balance: while higher LMWH doses may reduce occurrence of VTE, lower doses may reduce occurrence of bleeding.

Five small RCTs (total *N* = 412) have directly compared the effects of different LMWH types and doses in ICU patients *without* COVID‐19. However, their results were limited by imprecision or the use of surrogate outcomes [8, 9, 10]. Two of these RCTs assessed weight‐adjusted dosing and found a dose–response effect on anti‐Xa levels but were too small to reliably assess clinical outcomes [9, 11]. Increased body mass index (BMI) has been associated with thromboprophylaxis failure, suggesting potential underdosing in overweight patients when using fixed‐dose regimens [12]. However, most evidence supporting this association is observational and based on surrogate outcomes [9, 11, 12, 13, 14]. RCTs in ICU patients *with* COVID‐19 have assessed different LMWH dosing strategies [6, 15, 16, 17, 18, 19]. While their results do not support high dose LMWH for thromboprophylaxis, their results are uncertain with respect to the benefits and harms of low to intermediate doses [6, 15, 16, 17, 18, 19]. In addition, several factors limit the extrapolation of findings in patients *with* COVID‐19 to those *without*. Most RCTs had considerable uncertain results, used composite outcomes that included arterial events, or included heterogeneous treatment arms that combined LMWH and UFH [6, 16, 20, 21].

Major guidelines recommend pharmacological thromboprophylaxis for all ICU patients without contraindications [22, 23, 24]. Whereas the *European Society of Anaesthesiology and Intensive Care* guideline suggests an intermediate dose LMWH for prophylaxis in adult ICU patients (conditional recommendation, moderate certainty of evidence) [24], others provide no dose recommendations [22, 23]. A recent network meta‐analysis suggested that a fixed intermediate dose LMWH may confer the best balance between benefits and harms for the prevention of VTE compared to a low dose LMWH in acutely ill hospitalised patients, but the certainty of evidence was low due to imprecision and inconsistency resulting from between‐study heterogeneity; in addition, few ICU patients were included [25]. A survey among 715 intensivists from 170 ICUs across 23 countries reported considerable practice variation in LMWH dosing within and between ICUs [26]. The lack of evidence‐based dosing recommendations in guidelines, practice variation and competing risks of VTE and bleeding represent an unmet clinical need to assess the optimal prophylactic dosing strategy for LMWH in ICU patients [27].

The *INCEPT‐Thromboprophylaxis* domain will assess the effects of different doses of LMWH for thromboprophylaxis on days alive out of hospital at 30 days and other patient‐important outcomes including VTE and major bleeding events in adult, acutely admitted ICU patients. We hypothesise that a weight‐adjusted LMWH dose will be superior to both fixed low and fixed intermediate dose LMWH regarding days alive out of hospital at 30 days, and that the fixed low and intermediate dose LMWH will be practically equivalent.

## 2 Methods

### 2.1 Design, Approvals and Consent

The *INCEPT‐Thromboprophylaxis* domain is an investigator‐initiated, open‐label domain with an integrated feasibility phase on the pragmatic, parallel‐group, randomised, embedded, multifactorial, international, adaptive *Intensive Care Platform Trial (INCEPT)* [28]. The full core protocol and domain‐specific appendices for all approved domains are available at the trial website (www.incept.dk) [29]; these documents include additional details, variable definitions and completed reporting checklists [30, 31].

*INCEPT* and *INCEPT‐Thromboprophylaxis* have been approved by the Danish competent authorities and publicly registered before initiation (approvals and identifiers: EUCT number: 2024‐516208‐41‐00; ClinicalTrials.gov identifier: NCT06667999; Universal Trial Number: U1111‐1313‐8171); additional approvals will be obtained as required before initiation in other countries. Screening for *INCEPT‐Thromboprophylaxis* started on 18 September 2025.

*INCEPT* is an emergency medical trial generally enrolling participants without prior consent, followed by informed consent to continue in each domain from legal surrogates and participants as soon as possible; consent may be withdrawn at any time without explanation on a domain‐basis, as described in detail elsewhere [28, 29].

### 2.2 Eligibility Criteria

Adult (≥ 18 years) acutely admitted ICU patients will be screened for *INCEPT‐Thromboprophylaxis* if there is a decision by the treating clinician to start or continue thromboprophylaxis with LMWH, and all three dosing strategies are considered clinically appropriate. Patients will be excluded for the following reasons: legal consent expected to be unobtainable; under coercive measures; more than one prophylactic dose of anticoagulation administered during current ICU stay (including ICU days in other hospitals); known or suspected previous adverse hypersensitivity reaction to any type of heparin; known pregnancy; mechanical circulatory support; solid organ transplant during current hospital admission; weight < 40 kg; or inclusion in another interventional trial or *INCEPT* domain where co‐enrolment with *INCEPT‐Thromboprophylaxis* is not permitted. Patients may only be randomised once to *INCEPT‐Thromboprophylaxis*.

### 2.3 Interventions

The intervention period is from randomisation to a maximum of 90 days while in the ICU, including transfers or readmissions to participating ICUs during this period.

#### 2.3.1 LMWH Doses (Interventions)

The intervention is LMWH for thromboprophylaxis, administered in either a fixed low, fixed intermediate or weight (actual body weight at screening)‐adjusted dose (Table 1). Each trial site uses one of four LMWHs: dalteparin, enoxaparin, nadroparin or tinzaparin. To minimise the amount of within‐arm heterogeneity, we established the dose categorisation based on two principles:

1. *Alignment with clinical practice*: this was informed by survey results [26], national summaries of product characteristics, and the clinical experience of the domain management committee.
2. *Proportionality to the recommended therapeutic dose*: each LMWH has a well‐defined therapeutic weight‐adjusted dose, but prophylactic dosing lacks such standardisation. We therefore expressed prophylactic doses as percentages of therapeutic doses to achieve comparable dosing across LMWHs within each arm. For example, recommended dalteparin prophylactic doses are 2500 or 5000 IU once daily, while the therapeutic dose is 200 IU/kg once daily. For an 80 kg patient (therapeutic dose: 16,000 IU), prophylactic doses of 2500 and 5000 IU represent 15.6% and 31.3% of the therapeutic dose, respectively. These percentages were used to identify comparable doses for all four LMWHs types within the weight‐adjusted arm. Additionally, we applied rounding of doses to comply with contents available in syringes.

**TABLE 1:** Interventions.

Intervention	Weight (kg)	Enoxaparin dose (mg)	Tinzaparin dose (IU)	Dalteparin dose (IU)	Nadroparin dose (IU)
Fixed low dose	All	40	2500	2500	2850
Fixed intermediate dose	All	60	4500	5000	5700
Weight‐adjusted dose^a^	≥ 40–50	40	2500	2500	2850
	≥ 50–60	40	2500	2500	3800
	≥ 60–70	40	4500	5000	3800
	≥ 70–80	60	4500	5000	3800
	≥ 80–90	60	4500	5000	5700
	≥ 90–100	80	7000	7500	5700
	≥ 100–110	80	7000	7500	7600
	≥ 110–120	80	8000	7500	7600
	≥ 120	100	8000	10,000	7600

*Note: INCEPT‐Thromboprophylaxis* intervention arms.

Abbreviations: IU, international units; kg, kilogram; mg, milligram.

^a^ Actual body weight, measured or estimated at screening.

#### 2.3.2 Co‐Interventions

Co‐interventions will be at the discretion of the treating clinicians (except for randomised comparisons in other *INCEPT* domains or trials with approved co‐enrolment [28, 29]).

### 2.4 Outcomes

The primary outcome, also guiding all adaptations, is days alive out of hospital at 30 days, with non‐survivors assigned 0 days [21]. Secondary outcomes include the remaining *INCEPT* core outcomes and domain‐specific outcomes (VTE, major bleeding) including safety outcomes (severe anaphylactic reaction to LMWH, heparin‐induced thrombocytopenia, suspected unexpected serious adverse reactions) [17, 20] (Table 2).

**TABLE 2:** Outcome data.

Outcome	Outcome data by intervention^a^ descriptive data (*n*/*N* (%) or median [IQR]) model estimate (probability or mean [95% CrI])			Intervention effect estimates^b^ absolute difference [MD/RD (95% CrI)] relative difference (RoM/RR [95% CrI])			Probabilities of each intervention being best^c^		
	Low (*N* = #)	Int (*N* = #)	Weight‐adj. (*N* = #)	Low vs. int	Low vs. weight‐adj.	Int vs. weight‐adj.	Low	Int	Weight‐ adj.
Days alive out of hospital at Day 30^d^ (primary and guiding outcome)	*n*/*N* (#.#%) *#.#% (#.# to #.#)*	*n*/*N* (#.#) *#.#% (#.# to #.#)*	*n*/*N* (#.#) *#.#% (#.# to #.#)*	MD: #.## (#.## to #.##) *RoM: #.## (#.## to #.##)*	MD: #.## (#.## to #.##) *RoM: #.## (#.## to #.##)*	MD: #.## (#.## to #.##) *RoM: #.## (#.## to #.##)*	#.##%	#.##%	#.##%
Days alive without life support at Day 30^d^	#.# (#.# to #.#) *#.# (#.# to #.#)*	#.# (#.# to #.#) *#.# (#.# to #.#)*	#.# (#.# to #.#) *#.# (#.# to #.#)*	MD: #.## (#.## to #.##) *RoM: #.## (#.## to #.##)*	MD: #.## (#.## to #.##) *RoM: #.## (#.## to #.##)*	MD: #.## (#.## to #.##) *RoM: #.## (#.## to #.##)*	#.##%	#.##%	#.##%
Days free of delirium at Day 30^d^	#.# (#.# to #.##) *#.# (#.# to #.#)*	#.# (#.## to #.#) *#.# (#.# to #.#)*	#.# (#.# to #.#) *#.# (#.# to #.#)*	MD: #.## (#.## to #.##) *RoM: #.## (#.## to #.##)*	MD: #.## (#.## to #.##) *RoM: #.## (#.## to #.##)*	MD: #.## (#.## to #.##) *RoM: #.## (#.## to #.##)*	#.##%	#.##%	#.##%
All‐cause 30‐day mortality	#.# (#.# to #.##) *#.# (#.# to #.#)*	#.# (#.## to #.#) *#.# (#.# to #.#)*	#.# (#.# to #.#) *#.# (#.# to #.#)*	RD: #.## (#.## to #.##) *RR: #.## (#.## to #.##)*	RD: #.## (#.## to #.##) *RR: #.## (#.## to #.##)*	RD: #.## (#.## to #.##) *RR: #.## (#.## to #.##)*	#.##%	#.##%	#.##%
Days alive without life support at Day 90^d^	#.# (#.# to #.#) *#.# (#.# to #.#)*	#.# (#.# to #.#) *#.# (#.# to #.#)*	#.# (#.# to #.#) *#.# (#.# to #.#)*	MD: #.## (#.## to #.##) *RoM: #.## (#.## to #.##)*	MD: #.## (#.## to #.##) *RoM: #.## (#.## to #.##)*	MD: #.## (#.## to #.##) *RoM: #.## (#.## to #.##)*	#.##%	#.##%	#.##%
Days alive out of hospital at Day 90^d^	#.# (#.# to #.##) *#.# (#.# to #.#)*	#.# (#.# to #.#) *#.# (#.# to #.#)*	#.# (#.# to #.#) *#.# (#.# to #.#)*	MD: #.## (#.## to #.##) *RoM: #.## (#.## to #.##)*	MD: #.## (#.## to #.##) *RoM: #.## (#.## to #.##)*	MD: #.## (#.## to #.##) *RoM: #.## (#.## to #.##)*	#.##%	#.##%	#.##%
All‐cause 90‐day mortality	*n*/*N* (#.#%) *#.#% (#.# to #.#)*	*n*/*N* (#.#%) *#.#% (#.# to #.#)*	*n*/*N* (#.#%) *#.#% (#.# to #.#)*	RD: #.## (#.## to #.##) *RR: #.## (#.## to #.##)*	RD: #.## (#.## to #.##) *RR: #.## (#.## to #.##)*	RD: #.## (#.## to #.##) *RR: #.## (#.## to #.##)*	#.##%	#.##%	#.##%
All‐cause 180‐day mortality	*n*/*N* (#.#%) *#.#% (#.# to #.#)*	*n*/*N* (#.#%) *#.#% (#.# to #.#)*	*n*/*N* (#.#%) *#.#% (#.# to #.#)*	RD: #.## (#.## to #.##) *RR: #.## (#.## to #.##)*	RD: #.## (#.## to #.##) *RR: #.## (#.## to #.##)*	RD: #.## (#.## to #.##) *RR: #.## (#.## to #.##)*	#.##%	#.##%	#.##%
EQ‐5D‐5L index values [32] at Day 180^e^	#.## (#.## to #.##) *#.## (#.## to #.##)*	#.## (#.## to #.##) *#.## (#.## to #.##)*	#.## (#.## to #.##) *#.## (#.## to #.##)*	MD: #.## (#.## to #.##) *RoM: #.## (#.## to #.##)*	MD: #.## (#.## to #.##) *RoM: #.## (#.## to #.##)*	MD: #.## (#.## to #.##) *RoM: #.## (#.## to #.##)*	#.##%	#.##%	#.##%
EQ VAS [32] at Day 180^e^	#.# (#.# to #.#) *#.# (#.# to #.#)*	#.# (#.# to #.#) *#.# (#.# to #.#)*	#.# (#.# to #.#) *#.# (#.# to #.#)*	MD: #.## (#.## to #.##) *RoM: #.## (#.## to #.##)*	MD: #.## (#.## to #.##) *RoM: #.## (#.## to #.##)*	MD: #.## (#.## to #.##) *RoM: #.## (#.## to #.##)*	#.##%	#.##%	#.##%
Cognitive function (Mini MoCA) at Day 180^f^	#.# (#.# to #.#) *#.# (#.# to #.#)*	#.# (#.# to #.#) *#.# (#.# to #.#)*	#.# (#.# to #.#) *#.# (#.# to #.#)*	MD: #.## (#.## to #.##) *RoM: #.## (#.## to #.##)*	MD: #.## (#.## to #.##) *RoM: #.## (#.## to #.##)*	MD: #.## (#.## to #.##) *RoM: #.## (#.## to #.##)*	#.##%	#.##%	#.##%
Venous thromboembolism during hospital stay at Day 30^g^	*n*/*N* (#.#%) *#.#% (#.# to #.#)*	*n*/*N* (#.#%) *#.#% (#.# to #.#)*	*n*/*N* (#.#%) *#.#% (#.# to #.#)*	RD: #.## (#.## to #.##) *RR: #.## (#.## to #.##)*	RD: #.## (#.## to #.##) *RR: #.## (#.## to #.##)*	RD: #.## (#.## to #.##) *RR: #.## (#.## to #.##)*	#.##%	#.##%	#.##%
Venous thromboembolism during hospital stay at Day 90^g^	*n*/*N* (#.#%) *#.#% (#.# to #.#)*	*n*/*N* (#.#%) *#.#% (#.# to #.#)*	*n*/*N* (#.#%) *#.#% (#.# to #.#)*	RD: #.## (#.## to #.##) *RR: #.## (#.## to #.##)*	RD: #.## (#.## to #.##) *RR: #.## (#.## to #.##)*	RD: #.## (#.## to #.##) *RR: #.## (#.## to #.##)*	#.##%	#.##%	#.##%
Major bleeding during hospital stay at Day 30^h^	*n*/*N* (#.#%) *#.#% (#.# to #.#)*	*n*/*N* (#.#%) *#.#% (#.# to #.#)*	*n*/*N* (#.#%) *#.#% (#.# to #.#)*	RD: #.## (#.## to #.##) *RR: #.## (#.## to #.##)*	RD: #.## (#.## to #.##) *RR: #.## (#.## to #.##)*	RD: #.## (#.## to #.##) *RR: #.## (#.## to #.##)*	#.##%	#.##%	#.##%
Major bleeding during hospital stay at Day 90^h^	*n*/*N* (#.#%) *#.#% (#.# to #.#)*	*n*/*N* (#.#%) *#.#% (#.# to #.#)*	*n*/*N* (#.#%) *#.#% (#.# to #.#)*	RD: #.## (#.## to #.##) *RR: #.## (#.## to #.##)*	RD: #.## (#.## to #.##) *RR: #.## (#.## to #.##)*	RD: #.## (#.## to #.##) *RR: #.## (#.## to #.##)*	#.##%	#.##%	#.##%
One or more domain‐specific safety outcomes at Day 30^i^	*n*/*N* (#.#%) *#.#% (#.# to #.#)*	*n*/*N* (#.#%) *#.#% (#.# to #.#)*	*n*/*N* (#.#%) *#.#% (#.# to #.#)*	RD: #.## (#.## to #.##) *RR: #.## (#.## to #.##)*	RD: #.## (#.## to #.##) *RR: #.## (#.## to #.##)*	RD: #.## (#.## to #.##) *RR: #.## (#.## to #.##)*	#.##%	#.##%	#.##%
One or more domain‐specific safety outcomes at Day 90^i^	*n*/*N* (#.#%) *#.#% (#.# to #.#)*	*n*/*N* (#.#%) *#.#% (#.# to #.#)*	*n*/*N* (#.#%) *#.#% (#.# to #.#)*	RD: #.## (#.## to #.##) *RR: #.## (#.## to #.##)*	RD: #.## (#.## to #.##) *RR: #.## (#.## to #.##)*	RD: #.## (#.## to #.##) *RR: #.## (#.## to #.##)*	#.##%	#.##%	#.##%

*Note:* Mock table illustrating how we plan to present data from the final, primary analyses of all clinical outcomes in the *INCEPT‐Thromboprophylaxis* domain. All model‐based estimates, intervention effects and probabilities are based on the sample‐average posterior distributions for the outcome of interest. Definitions of all outcomes are provided in the core protocol and full domain‐specific appendix [29].

Abbreviations: CrI, credible interval; Int, intermediate dose low‐molecular‐weight heparin; Low, low dose low‐molecular‐weight heparin; MD, mean difference; *n* and *N*, *n* denotes number of participants with the outcome, *N* denotes the total number of participants in each arm; RD, (absolute) risk difference; RoM, ratio of means; RR, relative risk; Weight‐adj., weight‐adjusted dose low‐molecular‐weight heparin.

^a^ For all interventions, descriptive outcome data (counts with percentages for binary outcomes, medians with interquartile ranges for continuous/count outcomes) and estimated sample‐average probabilities (binary outcomes) or mean values (continuous/count outcomes) from the primary models will be presented with 95% CrIs. The counts and proportions of participants with missing data for each separate outcome variable will be reported, with descriptive data and model estimates based on multiply imputed data unless complete.

^b^ Sample‐average intervention effect estimates will be presented as all between‐arm comparisons.

^c^ The probabilities of each arm being the best will be presented; secondarily, the probabilities of each arm being superior compared to the other arms pairwise will be presented. Probabilities of practical equivalence between all arms and for all pairwise comparisons will also be presented (along with the probabilities of intervention effects larger than the practical equivalence threshold(s) in both directions for the pairwise comparisons). For all outcomes, the complete posterior distributions of the primary set of sample‐average intervention effects and the corresponding probabilities of *all* effect sizes will be visualised.

^d^ All *days alive without X*‐type outcomes will be reported with non‐survivors assigned 0 days and using actual values without penalisation of death in sensitivity analyses [33].

^e^ EQ‐5D‐5L index values will primarily be calculated using national value sets. Non‐survivors will be assigned 0 for EQ‐5D‐5L index values (corresponding to a health quality as bad as being dead) and EQ VAS (corresponding to the worst possible value), with secondary analyses conducted in survivors only.

^f^ Cognitive function assessed using the *Montreal Cognitive Assessment test 5‐min version, v2.1 (“Mini MoCA”)* [34], with non‐survivors assigned 0 (worst possible value) in the primary analyses and in survivors only in secondary analyses.

^g^ Venous thromboembolism during hospital stay includes any deep venous thrombosis of the lower or upper extremity or any pulmonary embolism registered in participating ICUs during hospital stay. Requesting diagnostic tests will be left to the discretion of the treating clinician. Systematic screening of all patients for DVT (i.e., regardless of clinical signs or symptoms) using compression ultrasound or any other imaging modality will not be allowed.

^h^ Major bleeding during hospital stay is defined as either a fatal bleeding, a symptomatic bleeding in a critical area or organ, such as intracranial, intraspinal, intraocular, retroperitoneal, intraarticular or pericardial, or intramuscular with compartment syndrome, or a bleeding that requires any of the following interventions: transfusion of ≥ 400 mL whole blood or red cells, endoscopy, endovascular management or surgery (open, laparoscopic, arthroscopic, endovascular).

^i^ One or more domain‐specific safety outcomes include severe anaphylactic reactions and heparin‐induced thrombopenia (none vs. possible/definite) in participating ICUs during hospital stay.

### 2.5 Randomisation and Allocation Concealment

Allocation will initially be equal and stratified for site and history of VTE (yes/no), followed by simple (unstratified) restricted response‐adaptive randomisation [35] after the first adaptive analysis with minimum 25% allocation to each arm (rescaled proportionally if one arm is dropped early) [28]. Allocation in *INCEPT‐Thromboprophylaxis* is independent of other active domains, and allocation concealment is secured via an electronic system available online.

### 2.6 Protocol Adherence, Feasibility and Separation

Protocol violations will be registered and centrally monitored. Treating clinicians may deviate from the protocol at any time to ensure participant safety if continued protocol adherence is assessed to lead to suboptimal care.

Dose adjustment or temporarily halting of the intervention is allowed in case of acute or chronic kidney injury, renal replacement therapy, thrombocytopenia, invasive procedures, use of thrombolysis and active (major) bleeding. The decision to adjust or withhold one or more doses of trial medication is at the discretion of the treating clinician. Suggested dose adjustment strategies are provided, but deviations are allowed.

An integrated feasibility phase including the first 300 participants will assess feasibility outcomes including separation (Table 3). The domain will continue without alterations if all feasibility criteria are met; otherwise, the domain management committee will decide if modifications are required.

**TABLE 3:** Feasibility outcomes.

Name	Definition/operationalisation	Feasibility criteria
Time to completion of the feasibility phase	Time to enrolment of the number of participants required for feasibility assessment	< 8 months
Recruitment proportion	Proportion of patients screened for *INCEPT‐Thromboprophylaxis* who are included in the domain	≥ 50%
Proportion of participants without consent to the continued collection of data	Proportion of participants for whom any consenting party do not consent to the continued collection of data (the number of participants for whom any consenting party do not consent to the continued collection of data divided by the number of randomised participants)	< 5%
Protocol adherence	Proportion of participants without protocol violations	≥ 75%
Retention proportion	Proportion of participants with data on the primary/guiding outcome within the outcome‐data lag period	≥ 95%
Separation between the fixed low dose and the weight‐adjusted dose arm	Proportion of participants in the weight‐adjusted arm who, according to the participant's weight at baseline and the type of LMWH used on the participant's first day of domain participation, were to receive a different dose than they would receive of the same type of LMWH in the fixed low dose arm	≥ 20%
Separation between the fixed intermediate dose and the weight‐adjusted dose arm	Proportion of participants in the weight‐adjusted arm who, according to the participant's weight at baseline and the type of LMWH used on the participant's first day of domain participation, were to receive a different dose than they would receive of the same type of LMWH in the fixed intermediate dose arm	≥ 20%

*Note:* Overview of feasibility outcomes, including definitions and feasibility criteria.

### 2.7 Statistical Methods

*INCEPT‐Thromboprophylaxis* will use Bayesian statistical methods [36] with neutral, weakly informative priors, adaptive stopping and response‐adaptive randomisation, and probabilistic interpretation of results [28].

#### 2.7.1 Analysis Sets and Primary Estimands

Analyses will primarily be conducted in the intention‐to‐treat population, with secondary analyses conducted in the per‐protocol population. The primary estimands in *INCEPT‐Thromboprophylaxis* are the sample‐average pairwise mean differences in the days alive out of hospital at 30 days between low, intermediate and weight‐adjusted dose LMWH in acutely admitted adult ICU patients with an indication for thromboprophylaxis with LMWH according to treatment allocation regardless of protocol adherence, with non‐survivors assigned 0 days.

#### 2.7.2 Adaptive Analyses and Adaptations

Adaptive analyses will be conducted after completion of follow‐up and data collection/validation for the primary outcome for the first 1500 participants and every 500 additional participants until a maximum sample size of 10,000 participants. Constant, symmetrical stopping rules for superiority/inferiority of 99.5% and 0.5% are used; these stopping rules have been calibrated using simulations to ensure a type 1 error rate (the probability of erroneously declaring one arm overall superior) below 5% for the primary outcome. The domain will be stopped for practical equivalence if there is > 90% probability that the mean difference in the primary outcome is < 1 day [35, 37]. Stopping rules are binding [38]. Response‐adaptive randomisation based on each arm's probability of being superior will be used after the first adaptive analysis, with a minimum allocation of 25% to each arm (rescaled to 37.5% if one arm is dropped) [28, 35, 36].

#### 2.7.3 Descriptive Statistics

Baseline data (Table 4), separation data and outcome data (Table 2) will be presented for each arm using medians with interquartile ranges for numeric data and counts with percentages for categorical data.

**TABLE 4:** Baseline data.

Characteristic	Fixed low dose LMWH (*N* = #)	Fixed intermediate dose LMWH (*N* = #)	Weight‐adjusted dose LMWH (*N* = #)
Country of enrolment			
Denmark	*n* (#.#%)	*n* (#.#%)	*n* (#.#%)
*[Each participating country listed separately in the domain report(s)]*	*n* (#.#%)	*n* (#.#%)	*n* (#.#%)
Age, median (IQR), years	## (## to ##)	## (## to ##)	## (## to ##)
Sex			
Female	*n* (#.#%)	*n* (#.#%)	*n* (#.#%)
Male	*n* (#.#%)	*n* (#.#%)	*n* (#.#%)
Weight, median (IQR), kg	## (## to ##)	## (## to ##)	## (## to ##)
Height, median (IQR), m	## (## to ##)	## (## to ##)	## (## to ##)
Use of invasive mechanical ventilation	*n* (#.#%)	*n* (#.#%)	*n* (#.#%)
Use of vasopressors/inotropes	*n* (#.#%)	*n* (#.#%)	*n* (#.#%)
Use of renal replacement therapy	*n* (#.#%)	*n* (#.#%)	*n* (#.#%)
Presence of a central venous catheter	*n* (#.#%)	*n* (#.#%)	*n* (#.#%)
Limitations of care	*n* (#.#%)	*n* (#.#%)	*n* (#.#%)
Co‐existing conditions			
Active haematological malignancy or metastatic cancer	*n* (#.#%)	*n* (#.#%)	*n* (#.#%)
History of ischaemic heart disease or heart failure	*n* (#.#%)	*n* (#.#%)	*n* (#.#%)
Diabetes mellitus	*n* (#.#%)	*n* (#.#%)	*n* (#.#%)
Chronic pulmonary disease	*n* (#.#%)	*n* (#.#%)	*n* (#.#%)
Chronic liver disease	*n* (#.#%)	*n* (#.#%)	*n* (#.#%)
Known use of immunosuppressive therapy within the last 3 months	*n* (#.#%)	*n* (#.#%)	*n* (#.#%)
Previous organ transplantation	*n* (#.#%)	*n* (#.#%)	*n* (#.#%)
Chronic dialysis	*n* (#.#%)	*n* (#.#%)	*n* (#.#%)
Treatment with antipsychotics at hospital admission	*n* (#.#%)	*n* (#.#%)	*n* (#.#%)
History of VTE	*n* (#.#%)	*n* (#.#%)	*n* (#.#%)
Suspected sepsis at ICU admission	*n* (#.#%)	*n* (#.#%)	*n* (#.#%)
Acute surgery within 7 days prior to randomisation	*n* (#.#%)	*n* (#.#%)	*n* (#.#%)
SMS‐ICU,^a^ median (IQR)	## (## to ##)	## (## to ##)	## (## to ##)
Predicted 90‐day mortality,^a^ median (IQR), %	##% (## to ##)	##% (## to ##)	##% (## to ##)
Predicted in‐hospital VTE risk,^b^ median (IQR), %	##% (## to ##)	##% (## to ##)	##% (## to ##)
Clinical Frailty Scale,^c^ median (IQR)	## (## to ##)	## (## to ##)	## (## to ##)
Lowest systolic blood pressure in the 24 h preceding randomisation, median (IQR), mmHg	## (## to ##)	## (## to ##)	## (## to ##)
Highest plasma lactate in the 24 h prior to randomisation, median (IQR), mmol/L	## (## to ##)	## (## to ##)	## (## to ##)
Highest plasma creatinine in the 24 h prior to randomisation, median (IQR), μmol/L	## (## to ##)	## (## to ##)	## (## to ##)
Lowest platelet count in the 24 h prior to randomisation, median (IQR), 10^9^/L	## (## to ##)	## (## to ##)	## (## to ##)

*Note:* Mock table illustrating how we plan to present baseline data. Binary or categorical variables are presented as numbers with percentages; numerical variables are presented as medians with interquartile ranges. Definitions of all baseline variables are provided in the core protocol [28] and full domain‐specific appendix [29].

Abbreviations: IQR, interquartile range; mmHg, millimetres of mercury; mmol/L, millimoles per litre; *N* or *n*, total counts or counts; SMS‐ICU, *Simplified Mortality Score for the Intensive Care Unit* [39]; μmol/L, micromoles per litre; VTE, venous thromboembolism.

^a^ SMS‐ICU [28] is a severity score (range 0–42 points) and prediction model, with higher values indicating more severe illness and higher risks of 90‐day mortality.

^b^ The predicted in‐hospital VTE incidence will be calculated using a modified version of the PROVE‐IT score [29].

^c^ Assessed by investigators using the Clinical Frailty Scale v2.0 [40] ranging from 1 (very fit) to 9 (terminally ill).

#### 2.7.4 Primary Analyses

All outcomes will be analysed using Bayesian linear and logistic regression models adjusted for the stratification variables (site and history of VTE) and the following additional baseline variables: age (modelled using a linear and a quadratic term), sex, haematologic malignancy or metastatic cancer, acute surgery within 7 days prior to randomisation, invasive mechanical ventilation, circulatory support, renal replacement therapy within 72 h prior to randomisation, and time period (with a new period defined after each adaptation) [28]. Analyses will primarily use neutral, weakly informative priors not favouring any intervention, with sensitivity analyses using neutral, more informative (sceptical) priors and neutral, less informative priors, as well as evidence‐based priors for outcomes with relevant external evidence [29]. Results will be presented as sample‐average estimates and intervention effects on the absolute and relative scales with the absolute summary measures being the primary [28], all calculated using G‐computation with the posterior probability distributions [29]. Point estimates, 95% credible intervals, and probabilities of each intervention being overall superior as well as probabilities of superiority in each comparison, and—for the primary outcome—probabilities of practical equivalence between all arms, and probabilities of practical equivalence and differences larger than the equivalence threshold in both directions for each comparison, will be reported as outlined in the core protocol [28]. Model fitting and model diagnostics are described in the full core protocol [28, 29].

#### 2.7.5 Heterogeneous Intervention Effects and Interactions

Once the domain is stopped, potential heterogeneous intervention effects for the primary outcome according to the baseline characteristics in Table 5 will be assessed on both the absolute and relative scales. For all analyses of heterogeneous intervention effects, results will be visualised with 95% credible intervals and interpreted probabilistically for all comparisons. No assessment of potential interactions with other domains is planned.

**TABLE 5:** Analyses of heterogeneity of intervention effects for the primary outcome.

Baseline characteristics	Definition and type	Expected direction of interaction
Disease severity	Baseline disease severity according to the Simplified Mortality Score for the Intensive Care Unit [39] (SMS‐ICU); continuous	Larger beneficial effects of the weight‐based dose compared to the other two doses in patients with more severe disease Similar effects of fixed low versus intermediate dose irrespective of severity
Risk of VTE	Baseline risk of VTE according to a modified version of the ‘Predicting the Risk of Venous Thromboembolism in Critically Ill Patients’ score [41] (PROVE‐IT); continuous	Larger beneficial effects of the weight‐based dose compared to the other two doses in patients with higher risk of VTE Similar effects of fixed low versus intermediate dose irrespective of VTE risk
Renal function	Highest plasma creatinine in the 24 h prior to randomisation; continuous	No substantial interactions between renal function and arms
Platelet count	Lowest platelet count in the 24 h prior to randomisation; continuous	No substantial interactions between platelet counts and arms

*Note:* Heterogeneity of intervention effects on the primary outcome will be analysed according to the baseline characteristics outlined in this table. The analyses of heterogeneity in intervention effects according to the continuous baseline variables listed above will be performed using linear regression models similar to the primary analysis model, but also including the baseline characteristic of interest, using linear and quadratic terms and their interactions with the intervention group (after mean‐centring and, for variables expected to be substantially right‐skewed, log 2‐transformation). Mean values for the primary outcome will be predicted in each intervention group across the full range of values of the baseline variable of interest using a G‐computation approach described in the full domain‐specific appendix [29] and used to calculate differences on the absolute and relative scales.

Abbreviations: PROVE‐IT, *Predicting the risk of venous thromboembolism in critically ill patients* [41]; SMS‐ICU, *Simplified Mortality Score for the Intensive Care Unit* [39]; VTE, venous thromboembolism.

#### 2.7.6 Missing Data Handling

Proportions of missing data will be presented, and missingness handled using multiple imputation with best‐worst/worst‐best case sensitivity analyses according to the core protocol [28, 29, 42].

#### 2.7.7 Statistical Simulation and Performance Metrics

The *INCEPT‐Thromboprophylaxis* domain design has been developed and evaluated using statistical simulation via R v4.5.0 (R Core Team, R Foundation for Statistical Computing, Vienna, Austria) with the *adaptr* R package (https://inceptdk.github.io/adaptr) following the approach previously described and used [35, 37, 43]. The final design as well as sensitivity analyses with different design choices and key assumptions were all evaluated using 100,000 simulations under multiple clinical scenarios. In brief, we assumed that the distribution of the primary and guiding outcome in the different doses of LMWH arms resembled the distribution from the *AFIB‐ICU* cohort study [44]. This reference distribution was modelled using a *binomial* distribution handling the probability of having 0 or > 0 days, and a *beta* distribution modelling the distribution among those with > 0 days. In summary, 43.6% had 0 days (22.3% due to death), with an estimated mean of 18 days in those with > 0 days, and an overall estimated mean of 10 days. Simulations were conducted in scenarios with no differences between arms, and with no, small and large differences in both directions between arms, corresponding to overall mean differences of 1 or 2 days in either direction. Performance metrics were assessed under a total of 15 realistic scenarios, constituting all unique combinations of the reference distribution in one arm and no/small/large differences in the other arms in both directions. Multiple performance metrics were evaluated, with the key metrics presented in Table 6. The expected sample sizes across scenarios ranged from 2265 to 4892 participants, with approximately 100% probabilities of conclusiveness across scenarios.

**TABLE 6:** Key performance metrics of the final domain design.

Metric	No differences	Ranges across scenarios with differences
Sample size—mean	4867	2265–4892
[25; 50; 75%‐percentiles]	[3950; 4450; 5450]	[1950–3450; 1950–4950; 2450–5950]
Mean number of days alive out of hospital at 30 days—mean	10.3 days	9.0–11.9 days
[25; 50; 75%‐percentiles]	[10.2; 10.3; 10.4]	[8.9–11.8; 9.0–11.9; 9.2–12.1]
Probability of conclusiveness	~100.0%	~100.0% to ~100.0%
Probability of superiority	0.9%	4.0%–99.9%
Probability of practical equivalence	99.1%	0.1%–96.0%
Probability of inconclusiveness	~0.0%	~0.0% to ~0.0%
Probability of erroneous superiority	0.1%	~0.0% to 4.6%
Root mean squared error (for estimated mean number of days in the superior arm, for simulations stopped for superiority only)	0.7 days	0.3–0.6 days
Ideal design percentage (for simulations stopped for superiority only)	—	100.0%–100.0%

*Note:* Key performance metrics of the final *INCEPT‐Thromboprophylaxis* domain design under the primary assumptions used based on 100,000 simulations for each scenario. The probabilities of conclusiveness correspond to the combined probabilities of superiority and practical equivalence; the probabilities of superiority may be interpreted as the type 1 error rate in the scenario with no difference and as the power in the scenarios with differences present. The probabilities of inconclusiveness refers to the proportion of simulated trials stopped at the maximum sample size without triggering any stopping rule. The probability of erroneous superiority refers to the probabilities of declaring one arm overall superior if it is not a single superior arm (i.e., if the arm is inferior to one or more other arms or if the arm is superior to one arm but equivalent to another arm). Ideal design percentages are calculated as described elsewhere; this measure cannot be calculated in the scenario where no differences between arms are present.

### 2.8 Stakeholder Involvement

Stakeholders were involved according to the requirements outlined in the core protocol [28, 29]. Nine ICU survivors, four family members, five nurses and two doctors not involved in planning or daily management of the domain were involved in discussions of domain outcomes including the primary/guiding outcome and provided inputs to the lay summary describing the domain and all written informed consent materials. Stakeholders were involved through established research panels via group discussions. Future stakeholder involvement is planned for the dissemination phase.

### 2.9 Organisational Aspects, Independent Data Monitoring and Safety Committee and Monitoring

Daily management of *INCEPT‐Thromboprophylaxis* is the responsibility of a domain sponsor and management committee, referring to the *INCEPT* platform sponsor and management committee. The domain is externally monitored according to the principles outlined in the core protocol [28, 29], and domain conduct and safety are overseen by a multidisciplinary independent data monitoring and safety committee [29].

### 2.10 Dissemination

We plan to present results from the integrated feasibility phase, for short‐term outcomes (30 days), and longer‐term (90–180 days) outcomes separately as preprints (for clinical outcomes), on the trial website, and in international peer‐reviewed medical journals, according to the *CONSORT‐ACE* guideline [31].

## 3 Discussion

The *INCEPT‐Thromboprophylaxis* domain compares the effects of fixed low versus fixed intermediate versus weight‐adjusted dose LMWH for thromboprophylaxis on days alive out of hospital at 30 days and other patient‐important outcomes in adult acutely admitted ICU patients. Due to the pragmatic design, large maximum sample size and the adaptive design, *INCEPT‐Thromboprophylaxis* will with high probability provide results directly informing clinical practice without continuing longer than necessary.

### 3.1 Strengths

The strengths of the *INCEPT* platform have been discussed elsewhere [28]; these also apply to *INCEPT‐Thromboprophylaxis*. This domain has several specific strengths. Its broad eligibility criteria allow for inclusion of high‐risk patients, including those with previous bleeding, thrombocytopenia or renal failure, which resembles daily clinical practice and optimises external validity. External validity is further promoted because the trial pragmatically evaluates the four most used LMWH types. By examining low‐dose, intermediate‐dose and weight‐adjusted LMWH regimens, the three intervention arms capture most of the existing clinical practice variation [26] and will address knowledge gaps in the current evidence base. Finally, the focus on VTE detected as part of routine clinical care (as opposed to trial‐specific screening procedures) captures clinically relevant events [26].

### 3.2 Limitations

*INCEPT‐Thromboprophylaxis* has limitations. The open‐label design may introduce a risk of bias. We consider this risk to be small for *hard* outcomes including the primary outcome of days alive out of hospital. However, there is a potential risk of bias in the domain‐specific secondary outcomes VTE and major bleeding. We cannot exclude that clinicians' knowledge of the intervention dose influences their decision to request imaging studies or temporarily halt the intervention around procedures. Also, clinicians may demonstrate a higher propensity to discontinue antiplatelet agents in participants receiving higher LMWH doses compared to those on lower doses. Although this has the potential to reduce internal validity, it may reflect real‐world practice. Fully blinding this domain would be costly, time‐consuming and logistically very challenging due to the different types and weight‐adjusted doses of LMWH and the numerous participating countries and sites. Therefore, we have chosen an open‐label design to enable recruitment of larger samples and thus increase the likelihood of providing precise results with higher overall probabilities of achieving conclusiveness. Second, the relatively small average difference in LMWH dose between the fixed dose arms and the weight‐adjusted arm raises the possibility that adequate separation may not be realised. This is assessed in the integrated feasibility phase that provides an opportunity to modify the protocol or update trial procedures if relevant. Third, the choice of primary outcome could be challenged, given the focus of previous thromboprophylaxis trials on VTE and major bleeding [5, 45]. We consider it likely that LMWH may affect the days alive out of hospital through its effects on VTE and major bleeding. These effects are expected to act in opposite directions, and the days alive out of hospital captures their combined impact. It is, therefore, more interpretable than a simple composite outcome. Also, the use of days alive out of hospital is in line with recent increases in the use of this outcome in other trials conducted in the critically ill [44], and recent recommendations to focus on more granular outcomes as these are both patient‐important [29] and provide more statistical information than mortality, increasing the probabilities of conclusive results [45].

## 4 Conclusions

The *INCEPT‐Thromboprophylaxis* domain will with high probability provide conclusive evidence on the comparative effectiveness of different doses of LMWH in adult acutely admitted ICU patients with an indication for thromboprophylaxis, measured by days alive out of hospital at 30 days and other patient‐important outcomes. This is expected to directly inform clinical practice.

## Author Contributions

Conceptualisation: all authors. Funding acquisition: Ruben Eck, Anders Granholm, Morten Hylander Møller, Anders Perner and Frederik Keus. Investigation: all authors. Methodology: all authors. Project administration: Ruben Eck, Anders Granholm, Aske Tøgern, Morten Hylander Møller and Anders Perner. Software: Anders Granholm and Aksel Karl Georg Jensen. Supervision: Anders Granholm, Morten Hylander Møller, Anders Perner and Frederik Keus. Writing – original draft: Ruben Eck, Anders Granholm, Anders Perner and Frederik Keus. Writing – review and editing: all authors.

## Funding

INCEPT is funded by the Novo Nordisk Foundation (NNF23OC0085106) and Sygeforsikringen ‘danmark’ (2020–0320), with additional support from Grosserer Jakob Ehrenreich og Hustru Grete Ehrenreichs Fond, Dagmar Marshalls Fond and Savværksejer Jeppe Juhl og hustru Ovita Juhls Mindelegat. The INCEPT‐Thromboprophylaxis domain is additionally funded by the Netherlands Thrombosis Foundation (2025_04). None of the funders have had any influence on the planning of the platform trial, and none will have any influence on the design, conduct, analysis or reporting of any domains assessed on the platform. None of the funders will have ownership of any trial data.

## Conflicts of Interest

The Department of Intensive Care at Rigshospitalet (A.G., M.H.M., A.P.) has received grants from the Novo Nordisk Foundation for other projects. Anders Perner received *research grants from Novo Nordisk Foundation, Sygeforsikringen ‘danmark’ and Savværksejer Jeppe Juhl*. Karina Meijer received *speaker fees from Alexion, participation in trial steering committees for Bayer and Astra Zeneca, consulting fees from Therini, participation in data monitoring and endpoint adjudication committee for Octapharma. All payments are to the institution*.

The other authors declare no conflicts of interest.

## Data Availability

No patient data were analysed in this protocol manuscript. Data sharing plans for the domain are described in the domain‐specific appendix and core protocol available at the trial website (www.incept.dk).
